# Losartan improved hippocampal long‐term potentiation impairment induced by repeated LPS injection in rats

**DOI:** 10.14814/phy2.14874

**Published:** 2021-05-27

**Authors:** Mahmoud Hosseini, Hossein Salmani, Yousef Baghcheghi

**Affiliations:** ^1^ Division of Neurocognitive Sciences, Psychiatry and Behavioral Sciences Research Center Mashhad University of Medical Sciences Mashhad Iran; ^2^ Neuroscience Research Center Mashhad University of Medical Sciences Mashhad Iran; ^3^ Applied Biomedical Research Center Mashhad University of Medical Sciences Mashhad Iran; ^4^ Student Research Committee Jiroft University of Medical Sciences Jiroft Iran

**Keywords:** angiotensin II, angiotensin II type 1 receptor blockers, inflammation, long‐term potentiation

## Abstract

Cognitive impairment has been known as a common consequence of brain inflammation. Long‐term potentiation (LTP), the generally accepted cellular mechanism for memory formation in the mammalian brain, has been shown to be suppressed by inflammation. Studies have shown that angiotensin II (Ang II) through the Ang II type 1 receptor (AT1R) has a role in brain and peripheral immune system communication and brain inflammation. Here, the effect of AT1R blockade on hippocampal LTP in rats undergoing repeated lipopolysaccharide (LPS) injection was investigated. Rats received intraperitoneal (ip) injections of LPS (250 μg kg^−1^ day^−1^) for seven days. Treatment with losartan (ip; 3 mg kg^−1^ day^−1^) was started 3 days before LPS injection and continued during the LPS injections. Rats were anesthetized, and field excitatory postsynaptic potential (fEPSP) was recorded from the *stratum radiatum* of the CA1 area of the hippocampus in response to stimulation of the Schaffer collateral pathway. Results showed that LTP was suppressed in the LPS‐injected rats as no significant differences were found in the fEPSP slope and amplitude before and after the LTP induction. AT1R blockade by losartan restored fEPSP to the control levels. These findings indicate that Ang II, through AT1R, has a role in LTP suppression induced by systemic inflammation.

## Graphical abstract

Rats received repeatedintraperitoneal injections of lipopolysaccharide (250 µg kg‐1 day‐1) for sevenconsecutive days while they were treated with losartan (3 mg kg‐1 day‐1).Long‐term potentiation (LTP) was recorded in the Schaffer collaterals‐CA1 synapses.LPS injection impaired hippocampal LTP as no significant differences wereobserved in fEPSP slope and amplitude before and after the LTP induction.Losartan significantly improved LTP impairment induced by LPS injection.

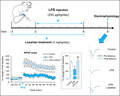

## 1 INTRODUCTION

Mounting evidence indicates that systemic inflammation has a significant role in the pathogenesis of brain diseases such as depression and anxiety (Miller & Raison, 2015) and memory failure (Sankowski et al., 2015; Skelly et al., 2019). In chronic inflammation, memory impairment may be present even after cessation of the initial stimulus of systemic inflammation (Salmani et al., 2021). Systemic bacterial endotoxin lipopolysaccharide (LPS) injection as a well‐characterized model of inflammation is commonly used to investigate the effects of peripheral inflammation on brain function (Brown, 2019). It has been reported that long‐term treatment of mice with LPS causes cognitive impairment, which persists beyond the acute effects of LPS injection (Lee et al., 2015; Salmani et al., 2021). In this study, repeated injections of LPS were used as a chronic model of systemic inflammation.

In the mammalian brain, long‐term potentiation (LTP) is the main accepted cellular mechanism underlying learning and memory (Bliss & Collingridge, 1993). Systemic inflammation has been reported to suppress LTP in the hippocampal CA1 area of experimental animals (Anaeigoudari et al., 2016; Chapman et al., 2010; Liu et al., 2012). While inflammatory cytokines in physiological levels have a pivotal role in healthy brain function, including learning and memory and LTP (Prieto & Cotman, 2017), in pathological conditions, inflammatory cytokines such as tumor necrosis factor‐α (TNF‐α) and interleukin (IL)‐1β can directly inhibit LTP in the hippocampus (Cunningham et al., 1996; Murray & Lynch, 1998; Prieto et al., 2019; Vereker et al., 2000).

The peripheral immune system communicates with the central nervous system (CNS) through three important routes, including cellular, neural, and humoral pathways (Holmes, 2013; Miller & Raison, 2015). Angiotensin‐II (Ang‐II) and its receptor Ang‐II type‐1 receptor (AT1R) have an important role in the communication of the peripheral immune system and CNS (Benicky et al., 2011; Saavedra, 2012). Furthermore, Ang‐II via AT1R acts as a pro‐inflammatory mediator in the CNS (Bhat et al., 2016). Additionally, it has been indicated that brain levels of Ang II and AT1R expression were increased in the LPS‐injected animals, as well as in glial cell culture exposed to LPS (Benicky et al., 2011; Bhat et al., 2016). Previous studies have shown that AT1R blockers (ARBs) prevent brain inflammation following systemic inflammation (Benicky et al., 2011; Salmani et al., 2020). Furthermore, Ang II directly modulates brain function, including processes related to learning and memory. For instance, intracerebroventricular (icv) injection of Ang II or renin has been shown to disrupt animal performance in the passive avoidance task, and this effect can be prevented by losartan, an AT1R blocker, and captopril, an angiotensin‐converting enzyme (ACE) inhibitor (DeNoble et al., 1991; Raghavendra et al., 1999). Studies also indicated that Ang II through the AT1R suppresses hippocampal LTP (Denny et al., 1991; von Bohlen und Halbach & Albrecht, 1998). Previous studies have shown that AT1R blockade by ARBs improves cognitive impairment in various models of brain inflammation (Khallaf et al., 2017; Quiñones et al., 2016; Villapol et al., 2015; Wincewicz & Braszko, 2015). Neuroprotective effects of losartan, as a selective AT1R blocker, in various models of brain disease have also been reported in previous studies (Salmani et al., 2020; Singh et al., 2017; Sun et al., 2015). In addition, in a mouse model of Alzheimer's disease, treatment with losartan has been reported to improve cerebrovascular function (Papadopoulos et al., 2017). While the protective effects of ARBs in inflammation‐induced memory impairment have been frequently reported, their effect on inflammation‐induced LTP suppression has been rarely investigated. In the present study, the protective effect of losartan on the inflammation‐induced LTP suppression resulting from repeated LPS injection was investigated.

## 2 MATERIALS AND METHODS

### 2.1 Reagents

Lipopolysaccharide (*E*. *coli*; serotype O55:B5) was purchased from Sigma Aldrich Chemical Co. Losartan (Batch. NO: LOS(04)‐16, Alborz Bulk pharmaceutical manufacturer) was a kind gift from PourSina Pharmaceutical Company.

### 2.2 Animals

The experiment was performed on 8–10 weeks old male Wistar rats (weighing 220–250 g) obtained from the animal house of Mashhad University of Medical Sciences. A week before starting the experiment, the animals were brought to the housing room to acclimatize to the new environment, and they were handled two min each day. The housing room had standard temperature conditions (23 ± 2℃) and light/dark cycle (12:12 h), and the animals had free access to rodent chow and water. The experimental procedure was approved by the Ethical Committee on Animal Research of Mashhad University of Medical Sciences (IR.MUMS.fm.REC.1394.562). All efforts were made to minimize the pain or discomfort of the animals.

### 2.3 Experimental design

**FIGURE 1:**
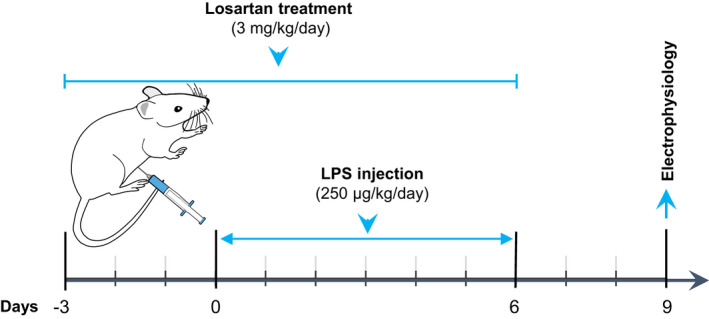
Experimental design. Rats received intraperitoneal injections of losartan (3 mg/kg) from day 3 until day 6 (10 days). LPS injections (250 μg/kg) were started from day 0 and continued until day 6 (7 days). Three days after the last LPS injection (day 9), rats were anesthetized and used for the electrophysiological experiment. LPS, lipopolysaccharide

An illustration of the experimental design is shown in Figure 1. Briefly, 22 rats were divided into three groups, including control (*n* = 8), LPS (*n* = 7), and LPS‐Losartan (*n* = 7). Rats received intraperitoneal (ip) injections of LPS (250 µg kg^−1^ day^−1^) or endotoxin‐free saline for seven consecutive days. LPS was dissolved in sterile saline at a concentration of 0.25 mg/ml and injected into the rats based on their weights. The dose of LPS and duration of injection were selected based on previous reports indicating that in this paradigm, LPS induces mild and chronic systemic inflammation (Lee et al., 2012; Salmani et al., 2021). Chronic treatment with losartan (ip, 3 mg kg^−1^ day^−1^) or its vehicle (sterile saline, 1 ml/kg) was started 3 days before the LPS injection and continued during the LPS injections. On each day, losartan was injected 30 min before the LPS injection. Losartan was dissolved in pathogen‐free saline and stored at 4℃. The dose and duration of treatment with losartan were selected based on previous studies (Koh et al., 2013; Salmani et al., 2018). At 72 h after the last LPS injection, rats were anesthetized and used for the electrophysiological experiment. This time point (72 h after the LPS injection) was chosen since the acute effects of LPS have been shown to diminish at this time point (Godbout et al., 2008; Muccigrosso et al., 2016),

### 2.4 Electrophysiology

#### 2.4.1 Surgery

**FIGURE 2:**
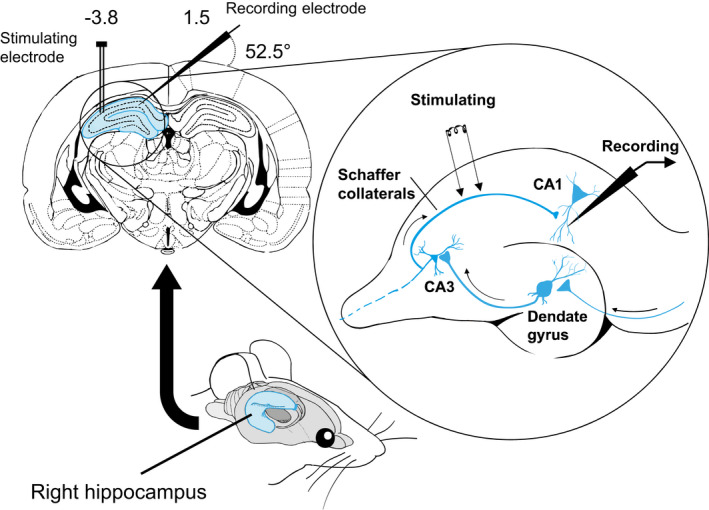
Long‐term potentiation recording in the Schaffer collateral‐CA1 synapses. A bipolar stimulating electrode was positioned in the Schaffer collateral pathway of the right hippocampus (coordinates; AP = −3.4 mm, ML = −3.8 mm, DV = 2.7‒3.8 mm) and recording electrode was lowered from the left side of the skull to the ipsilateral CA1 area (coordinates; AP = −4.2 mm, ML = 1.5, DV = 4.4‒5.1, at an angle of 52.5°). Monosynaptic field excitatory postsynaptic potentials (fEPSP) evoked by Schaffer collateral pathway stimulation were recorded in the CA1 area of the hippocampus

Monosynaptic field excitatory postsynaptic potentials (fEPSP) evoked by Schaffer collateral pathway stimulation were recorded in the CA1 area of the hippocampus. Schaffer collateral pathway‐CA1 synapses were used to study LTP since previous studies have reported that LPS injection suppresses the LTP induction in this area (Abareshi et al., 2016; Anaeigoudari et al., 2016; Strehl et al., 2014). Rats were anesthetized with urethane injection (ip; 1.6 g/kg), and their head was fixed in a stereotaxic frame. Then the animal skull was exposed, and CA1 and Schaffer collateral coordinates were determined on the skull according to the atlas of Paxinos and Watson (2007). The recording electrode was lowered from the left side of the skull and positioned in the right CA1 *stratum radiatum* (coordinates; AP = −3.4 mm, ML = 1.5, DV = 4.4‒5.1, at an angle of 52.5°) and a bipolar stimulating electrode was positioned in the ipsilateral Schaffer collateral pathway (coordinates; AP = −4.2 mm, ML = −3.8 mm, DV = 2.7‒3.8 mm; Figure 2; Sadeghi et al., 2017). The electrodes were made of 125 μm Teflon‐coated stainless steel wire (A‐M systems). A test recording procedure was conducted to determine the final position of the electrodes in which a reliable fEPSP with typical characteristics in the *stratum radiatum* layer of the hippocampal CA1 (Manahan‐Vaughan, 2018; Sweatt, 2008) was recorded.

#### 2.4.2 Stimulating and recording procedure

A two‐channel Electromodule amplifier (R12, ScienceBeam) was used to record the extracellular fEPSPs. The signals from the recording electrode were amplified (1000×), low‐pass filtered (1 Hz–3 kHz), digitized, and analyzed using the Electromodule R12 amplifier and eProbe software (Science Beam). Input–output (I/O) curves were constructed using stepwise increases of presynaptic fiber stimulation until the fEPSP amplitude was saturated. Then, for each animal, the stimulation strength was adjusted to give an fEPSP amplitude of 50% of the maximum response and kept constant at this level throughout the experiment. Before inducing LTP, stable baseline synaptic responses were recorded for 30 min, and then a high‐frequency stimulus (HFS; 100 pulses delivered at 100 Hz) was used to induce LTP. Synaptic responses were recorded 90 min following LTP induction (Anaeigoudari et al., 2016; Atabaki et al., 2020).

#### 2.4.3 Data analysis

The eProbe software (Science Beam Institute) was used for the off‐line analysis of fEPSP slope and amplitude. LTP was assessed as the increase in the amplitude and slope of the fEPSP (i.e., the 10%–90% rise time of the slope automatically calculated by the software) over the 90 min post‐tetanus period. Data were averaged over 5‐min intervals and expressed as the percentage change from baseline (mean ± *SEM*). Statistical analyses of data were done using the SPSS 26.0 software (SPSS Inc.). Paired sample *t*‐test or mixed‐design ANOVA (with treatment as between‐subjects factors) followed by Bonferroni post hoc test were used for statistical analyses of data. *p*‐values < 0.05 were considered as a significant difference between the mean values.

## 3 RESULTS

**FIGURE 3:**
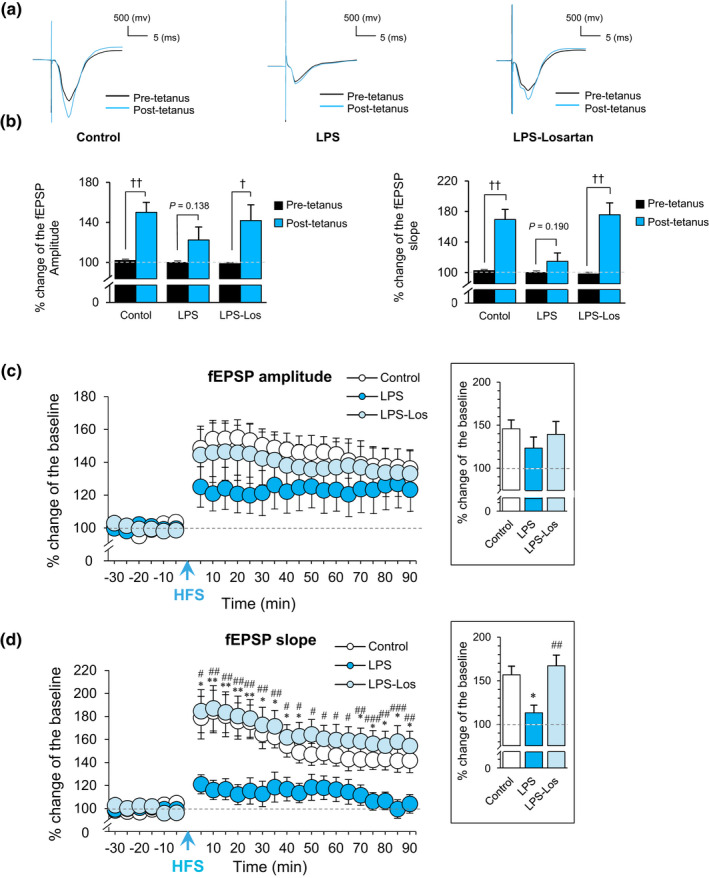
Losartan treatment reversed inflammation‐induced impairments in long‐term potentiation. (a) Representative pre‐ and post‐tetanus traces in Control, LPS, and LPS‐Los groups. (b) Percentage change of fEPSP amplitude and fEPSP slope pre and post‐tetanus (the normalized average of fEPSP amplitude and fEPSP slope in 15‐min pre‐tetanus and 20 to 40‐min post‐tetanus). Data were analyzed by paired sample *t*‐test. ^†^ *p* < 0.05 and ^††^ *p* < 0.01 shows the differences between pre‐tetanus and post‐tetanus fEPSP. (c) Percentage change in fEPSP amplitude and (d) slope. Data were analyzed by mixed‐design ANOVA followed by Bonferroni multiple comparison test. Bar graphs on the right side show the average percentage change of fEPSP in 90 min after the LTP induction. Data were analyzed by one‐way ANOVA followed by Tukey's post hoc test. *p < 0.05 and **p < 0.01 compared to the control group; ^#^ *p* < 0.05, ^##^ *p* < 0.01 and ^###^ *p* < 0.01 compared to the LPS group. Los, losartan; LPS, lipopolysaccharide; HFS, high‐frequency stimulation

To examine the influence of losartan treatment on synaptic transmission in the LPS‐treated rats, fEPSP was recorded at CA1 *str. radiatum* in response to stimulation of the Schaffer‐collaterals pathway (Figure 2). To measure LTP, the averaged fEPSP amplitude and slope during the final 15 min of pre‐tetanus baseline recording was compared with 20–40 min post‐tetanus recording. As expected, in the control group, the comparison of fEPSP amplitude (150 ± 9.95, paired sample *t*‐test, *t*_(7)_ = −4.89, *p* < 0.01) and slope (169.64 ± 13.1, paired sample *t*‐test, *t*_(7)_ = −4.97, *p* < 0.01) showed that LTP was successfully induced (Figure 3b). Interestingly, in the LPS group, LTP was not induced, as no significant differences were observed in fEPSP amplitude (122.42 ± 12.92, paired sample *t*‐test, *t*_(6)_ = −1.71, *p* = 0.138) and slope (114.7 ± 10.9, paired sample *t*‐test, *t*_(6)_ = −1.47, *p* = 0.190) between the pre‐tetanus and post‐tetanus recording. Pre‐treatment with losartan restored the post‐tetanus fEPSP amplitude (141.75 ± 15.7, paired sample *t*‐test, *t*_(6)_ = −2.61, *p* < 0.05) and slope (175.7 ± 15.52, paired sample *t*‐test, *t*_(6)_ = −4.679, *p* < 0.01) to the control level (Figure 3a,b).

To investigate the effect of losartan pretreatment on the magnitude of LTP in the LPS‐treated rats, fEPSP amplitude and slope were compared between the groups (Figure 3c,d). Analyzing of data with mixed‐design ANOVA revealed significant main effect of time (*F*_(23,437)_ = 25.45, *p* < 0.001), treatment (*F*_(2,19)_ = 7.29, *p* < 0.01), and time × treatment interaction (*F*_(46,437)_ = 3.4, *p* < 0.001) on fEPSP slope. There was a significant main effect of time (*F*_(23,437)_ = 18.84, *p* < 0.001) on fEPSP amplitude, but no significant effects of treatment (*F*_(2,19)_ = 0.831, *p* = 0.451) or time × treatment interaction (*F*_(46,437)_ = 0.951, *p* = 0.565). Further analysis of slope data with Bonferroni post hoc test showed that in the LPS group, fEPSP slope significantly decreased compared to the control rats (*p* < 0.05) and pretreatment with losartan restored the fEPSP slope to the control level (*p* < 0.01; Figure 3c,d).

## 4 DISCUSSION

It has been well known that inflammation impairs synaptic plasticity in various synapses of the CNS. Previous reports indicated that systemic inflammation induces a mirror inflammation in the brain and subsequently impairs multiple aspects of brain function, including hippocampal synaptic plasticity (Anaeigoudari et al., 2016; Di Filippo et al., 2013; Strehl et al., 2014). Although we did not evaluate cognitive function in this study, previous studies reported that repeated LPS injection induces spatial memory, recognition memory, and fear memory impairment (Lee et al., 2012; Salmani et al., 2021). In this study, we measured the protective effects of losartan on hippocampal LTP impairment induced by repeated LPS injection. Our findings indicated that LTP impairment in the Schaffer collateral‐CA1 synapses persisted beyond the acute effects of LPS injection. Consistent with these findings, it has been previously reported that tetanic stimulation of the Schaffer collateral pathway was not able to induce LTP in the Schaffer collateral‐CA1 synapses in LPS‐treated animals (Anaeigoudari et al., 2016; Di Filippo et al., 2013). On the other hand, it has been reported that multiple systemic LPS injections (twice a week for 1 month) impaired hippocampal LTP in Complete Freund's Adjuvant‐injected mice but not in normal mice (Maggio et al., 2013).

Our previous studies showed that IL‐1β and TNF‐α levels in the brain tissue of repeated LPS‐injected mice were significantly higher than the control animals even 2 weeks after LPS withdrawal (Salmani et al., 2020, 2021). In physiological concentrations, IL‐1β enhances hippocampal LTP and memory function, whereas, in pathological concentrations, it suppresses LTP and induces memory impairment (Goshen et al., 2007; Ross et al., 2003). Inflammatory cytokines have been reported to suppress LTP in hippocampal slices directly. Most importantly, two cytokines, including IL‐1β and TNF‐α, have been reported to impair LTP and cognitive function in experimental animals (Cunningham et al., 1996; Murray & Lynch, 1998; Vereker et al., 2000). However, cytokines start complex downstream events by inducing microglial activation, producing multiple inflammatory mediators, and altering regulatory proteins such as neurotrophins. For instance, it has been reported that while IL‐1β and TNF‐α directly suppress hippocampal LTP at synapses, IL‐18 impairs LTP by indirect mechanisms (Prieto et al., 2019). Indeed, studies suggested that IL‐1β is the final effector for most cytokines modulating LTP and memory (Prieto & Cotman, 2017). In this study, we did not evaluate the inflammatory cytokines in the brain tissue; however, based on previous reports, elevated pro‐inflammatory cytokines may be the reason for LTP suppression.

In our previous studies, we have shown that pretreatment with losartan prevented brain inflammation and cognitive impairment in LPS‐treated mice (Salmani et al., 2020) and rats (Salmani et al., 2018). In the present study, we further investigated the protective effect of losartan on hippocampal LTP following repeated systemic inflammation. Our results showed that pretreatment with losartan reversed hippocampal LTP impairment induced by systemic inflammation. There is considerable evidence regarding the effects of AT1R activation in suppressing LTP (Denny et al., 1991; von Bohlen und Halbach and Albrecht, 1998; Wright & Harding, 2004; Wright et al., 2002); however, the protective actions of ARBs on synaptic plasticity in pathological conditions like inflammation have been rarely investigated (Takeda et al., 2009). Strong evidence indicates that Ang II suppresses LTP, while Ang IV facilitates LTP (Wright et al., 2002). In addition, Ang II, through AT1R activation, contributes to the peripheral immune system and CNS communication. Previous studies have shown that AT1R blockade following systemic inflammation ameliorates brain inflammation (Benicky et al., 2011; Saavedra, 2012). Our previous study also showed that losartan prevented brain inflammation by reducing the inflammatory cytokines IL‐1β and TNF‐α in hippocampal tissues (Salmani et al., 2020). Both of these cytokines are highly expressed in a diseased brain, and both of them have been reported to directly suppress hippocampal LTP (Prieto et al., 2019). Therefore, losartan maybe by reducing the brain inflammation, was able to improve LTP. Moreover, it has been suggested that the blocking of AT1R leads to the conversion of excess endogenous Ang II to Ang IV in the brain, which in turn activates AT4R (Wright & Harding, 2013). It has been shown that Ang IV facilitates memory and LTP (Wright et al., 2002). However, more investigations are needed to be done to find the exact mechanism(s).

As a limitation of the study, we did not measure inflammatory mediators in the brain or periphery to attribute the beneficial effects of losartan to its anti‐inflammatory effects. The second limitation of the study was that the control group treated with losartan was not included in the experiment to examine the impact of losartan on LTP in normal conditions.

## 5 CONCLUSION

In conclusion, the findings of the present study demonstrate that repeated LPS injections suppress hippocampal LTP, and AT1R blockade by losartan can prevent LTP suppression resulting from prolonged systemic inflammation.

## CONFLICT OF INTEREST

None.

## AUTHOR CONTRIBUTION

Mahmoud Hosseini: Conceptualization, Methodology, Validation, Resources, Data curation, Writing – review and editing, Supervision, Project administration, Funding acquisition. Hossein Salmani: Conceptualization, Methodology, Formal analysis, Investigation, Data curation, Writing – original draft, Visualization. Yousef Baghcheghi: Writing – review and editing, Investigation.
